# Effect of Elderberry (*Sambucus nigra* L.) Extract Supplementation in STZ-Induced Diabetic Rats Fed with a High-Fat Diet

**DOI:** 10.3390/ijms18010013

**Published:** 2016-12-22

**Authors:** Ângelo C. Salvador, Ewelina Król, Virgínia C. Lemos, Sónia A. O. Santos, Fernanda P. M. S. Bento, Carina P. Costa, Adelaide Almeida, Dawid Szczepankiewicz, Bartosz Kulczyński, Zbigniew Krejpcio, Armando J. D. Silvestre, Sílvia M. Rocha

**Affiliations:** 1 Organic Chemistry, Natural Products and Food Stuffs Research Unit, QOPNA, Department of Chemistry, University of Aveiro, 3810-193 Aveiro, Portugal; angelomcsalvador@ua.pt (Â.C.S.); virginia.lemos@ua.pt (V.C.L.); fernandasaobento@ua.pt (F.P.M.S.B.); 2 Aveiro Institute of Materials, CICECO, Department of Chemistry, University of Aveiro, 3810-193 Aveiro, Portugal; santos.sonia@ua.pt (S.A.O.S.); armsil@ua.pt (A.J.D.S.); 3 Human Nutrition and Hygiene, Poznan University of Life Sciences, Wojska Polskiego 31, 60-637 Poznan, Poland; ekrol@up.poznan.pl (E.K.); bartekk@up.poznan.pl (B.K.); zkre@up.poznan.pl (Z.K.); 4 Department of Biology, University of Aveiro, 3810-193 Aveiro, Portugal; carina.pedrosa@live.ua.pt; 5 Centre for Environmental and Marine Studies, CESAM, Department of Biology, University of Aveiro, 3810-193 Aveiro, Portugal; aalmeida@ua.pt; 6 Department of Animal Physiology and Biochemistry, Poznan University of Life Sciences, Wojska Polskiego 35, 60-637 Poznan, Poland; dawszczepankiewicz@gmail.com

**Keywords:** diabetic rats, elderberry extracts supplementation, high-fat diet, lipophilic extract, polar extract, *Sambucus nigra* L.

## Abstract

Elderberry (*Sambucus nigra* L.) lipophilic and polar extract dietary supplementation effects were evaluated according to diabetes management indices, using an in vivo model. A research pipeline was constructed, that ranged from extract preparation, partial chemical characterization and toxicity evaluation, to examining the elderberry extract dietary supplementation effects on biofluid and tissues. Extracts toxicity was screened using an *Aliivibrio fischeri* bioluminescence model. A concentration of up to 60 mg/L was selected, and rat doses for oral supplementation were computed applying the interspecies correlation between *A. fischeri* and rats. Wistar type 2 diabetic rats, induced by streptozotocin (STZ), were fed a high-fat diet and supplemented for 4 weeks at doses of 190 and 350 mg/kg body weight/day of lipophilic and polar extract, respectively. As far as we know, lipophilic elderberry extract supplementation was assessed for the first time, while polar extract was administrated at higher doses and for a shorter period compared to previous studies, aiming to evaluate subacute supplementation effects. The polar extract modulated glucose metabolism by correcting hyperglycemia, while the lipophilic extract lowered insulin secretion. Both extracts lowered insulin resistance, without remarkable alterations to hematological indices, sera lipids and sera and tissular trace element homeostasis. In conclusion, elderberries are a potential source of bioactive compounds for formulations to be used as co-adjuvants in diabetes management.

## 1. Introduction

Diabetes mellitus is a multiple etiology metabolic disorder with abnormalities in the metabolism of carbohydrate, fat and protein, being characterized by chronic hyperglycemia and defects in insulin secretion, insulin action, or both [1]. It is projected that diabetes will affect over 300 million people by the year 2030 [2,3,4]. The relevance of this disorder, namely its social impact, is clearly highlighted by the World Health Organization (WHO) which proposes that further research is urgently needed to evaluate the effectiveness of interventions to prevent it, including behavioral changes, favoring a diet with increased fruit and vegetable consumption and, thus, improving dietary patterns [2]. Diets or food supplements that contribute to control and/or prevent hyperglycemia might be crucial to reducing diabetes incidence [5], namely through the development of alternative sources of antidiabetic agents.

European elderberry (*S. nigra* L.) is a deciduous shrub that produces violet-black drupes which grow in clusters, holding hundreds of berries [6]. This plant is considered from the days of Hippocrates as the “medicine chest” [7], and has been used in the formulation of diverse medicinal preparations to prevent and/or control different diseases [8]. Several bioactive compounds are reported on elderberries, namely phenolic compounds as anthocyanin derivatives, including cyanidin 3-glucoside, cyanidin 3-sambubioside, cyanidin 3-sambubioside-5-glucoside and cyanidin 3,5-diglucoside [9,10,11]; as well as triterpenic compounds such as ursolic and oleanolic acids, and sterols, as β-sitosterol were reported as elderberry bioactive components [12,13].

The huge importance of searching for alternative sources of antidiabetic agents and the limited number of studies dealing with elderberry extract supplementation to reduce diabetes complications, highlights the need to conduct more detailed studies on this topic. Previous studies revealed the potential of elderberry extracts in diabetes status management [5,14,15,16,17,18,19]. The effects of acidified methanol elderberry extracts dietary supplementation (28–70 mg of extract/kg body weight (b.w.) streptozotocin (STZ)-induced diabetic Wistar rats, during 12 to 16 weeks) were evaluated showing a reduction in serum glycemic and lipidic levels (cholesterol and triacylglycerol); reduction in the levels of oxidative markers (as superoxide dismutase and glutathione peroxidase activities) and inflammatory markers (as interleukin-6); and an increase in immunological parameters from T lymphocytes populations [5,14,15,16,17,18,19]. The metabolic effects of elderberry extract supplementation in an obese mouse model C57BL/6J were also evaluated (20–200 mg of extract/kg b.w., fed either with a low-fat diet and high-fat diet, for 16 weeks), and decreased serum triacylglycerol, inflammatory markers (as TNF-α) and insulin resistance were reported [20]. Despite the claim that these effects were attributable to phenolic components [5,14,15,16,17,18,19], structure–activity relationship studies were not conducted. Furthermore, only a few in vivo studies were performed, in which diets were supplemented with bioactive components present in elderberries, such as cyanidin 3-glucoside, quercetin 3-rutinoside and ursolic acid. Cyanidin 3-glucoside dietary supplementation (0.2% of the diet during 5 weeks) promoted a reduction in the blood glucose levels and an enhancement of insulin sensitivity in type 2 diabetic KK-A^y^ mice [21]; quercetin 3-rutinoside supplementation (25–100 mg/kg during 45 days) revealed antihyperglycemic and antioxidant activity on STZ-injected Wistar rats [22]; while ursolic acid supplementation lowered the urine excretion and renal oxidative stress levels in STZ-injected Wistar rats (0.2% of the diet during 16 weeks) [23]. The fact that elderberry extracts and their major components are linked with different health benefits including preventing diabetes complications warrants considering the use of elderberry enriched extracts to access their potential antidiabetic effect.

It is worth noting that any compound that interferes with a biological system might raise toxicity concerns, thus a screening of plant extract toxicity before in vivo assays is of major importance. Different models are currently used for this purpose; namely based on the use of microorganisms. *Aliivibrio fischeri* bioluminescence method is widely used to evaluate the toxicity response as it correlates bioluminescence signal and viable counts, where light output reflects the cells’ metabolic rate being therefore a rapid, sensitive and cost-effective option [24,25]. Disturbances in the bacterial metabolism implies alterations to light production, as the *A. fischeri* cellular respiration and light emission metabolic pathway are intrinsically linked [26].

From this perspective, this study aims to evaluate the *S. nigra* L. lipophilic (dichloromethane) and polar (acidified methanol) extract dietary supplementation effects on an animal model of diabetes, in order to obtain insights on their effects on diabetes and related complications. Wistar streptozotocin (STZ)-induced diabetic rats fed with a high-fat diet were enrolled in this study, and non-diabetic and diabetic rats without supplementation were also used as controls. Hematological and biochemical blood indices, as well as blood and tissular trace elements were assessed. A specific set of biochemical parameters were analyzed, namely fasting blood glucose and insulin, as there have been increasing efforts in search of bioactive compounds or extracts that can improve insulin action and lower blood glucose levels [27]. Furthermore, type 2 diabetes allied with a high fat diet regimen might induce changes in lipidic patterns, as well as hepatic dysfunctions, highlighting the need to understand whether elderberry extract supplementation might improve these conditions. In order to obtain fractions enriched in lipophilic and polar bioactive components (e.g., in triterpenic acids and in phenolic compounds), dietary elderberry supplementations were performed using extracts instead of whole elderberries. Thus, firstly the polar extract was characterized by ultra-high-pressure liquid chromatography-tandem mass spectrometry (UHPLC-MS*^n^*) analysis, while lipophilic fraction characterization by gas chromatography-mass spectrometry (GC-MS) was reported elsewhere [13]. Secondly, elderberry extract toxicity was evaluated using the bioluminescence *A. fischeri* assay. Diets were then prepared and administrated to rats using doses selected based on the preliminary toxicity assays.

## 2. Results and Discussion

**Figure 1:**
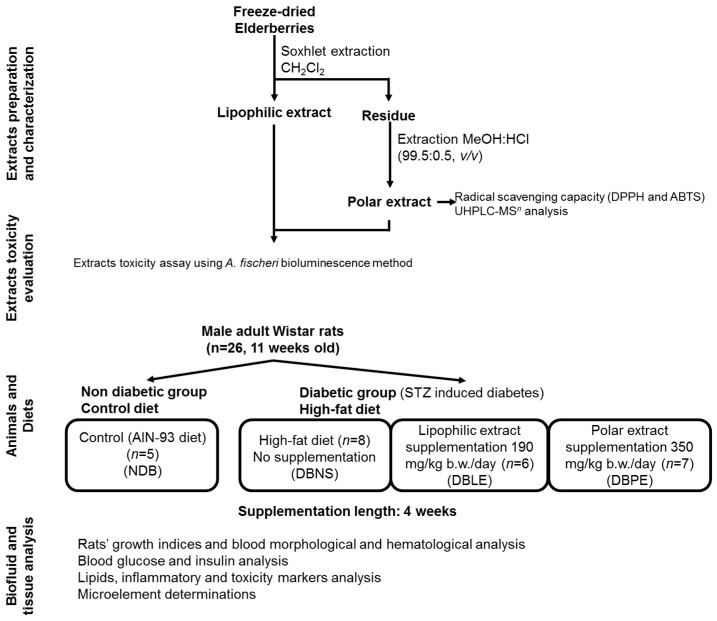
Main stages of elderberry extract preparation, partial characterization, and toxicity evaluation; followed by Wistar rat elderberry extract supplementation and biofluid and tissue analysis. NDB: non-diabetic group; DBNS: diabetic group/not supplemented; DBLE: diabetic group/supplemented with lipophilic extract; and DBPE: diabetic group/supplemented with polar extract. UHPLC-MS*^n^*: ultra-high-pressure liquid chromatography-tandem mass spectrometry, DPPH: 2,2-diphenyl-1-picrylhydrazyl, ABTS: 2,2′-Azino-bis(3-ethylbenzothiazoline-6-sulphonic acid).

The strategy followed in this work (Figure 1) included the preparation of the two extracts from freeze-dried berries, followed by their characterization, and toxicity evaluation to define non-toxic doses, and finally administration to rats. The effects of the elderberry extract dietary supplementation on high-fat fed diabetic Wistar rats were assessed in a biofluid and tissue analysis. Diabetic rats without elderberry dietary supplementation and non-diabetic rats were also followed-up.

### 2.1. Elderberry Extract Preparation, Radical Scavenging Activity and UHPLC-MS^n^ Analysis

The dichloromethane Soxhlet extraction yield was 1.69% ± 0.25% (m/m, d.w.), being within the values previously reported for this matrix [13]. The detailed composition of this extract was previously reported [13], showing that triterpenic acids, namely ursolic and oleanolic acids, were detected as the major components (ca. 85%–90% of the identified compounds), followed by fatty acids, fatty alcohols and sterols. The overall content of the identified compounds in elderberry dichloromethane extracts ranged from 19.6 to 26.4 g/100 g extract, with overall values of triterpenic compounds from 16.3 to 22.3 g/100 g extract.

The yields of the polar extract, obtained after removal of the lipophilic components (Figure 1) and the antioxidant activity, obtained by the DPPH and ABTS assays, are shown in Table 1. The extraction yield (58.2% m/m, d.w.) was similar to previously reported values (60.2%) for *S. nigra* berries using the same solvent [28]. Elderberry polar extract showed ABTS and DPPH radical scavenging activities of 2.37 and 0.63 mmol trolox equivalents (TE)/g extract, respectively. These values are in agreement with previously published data for aqueous elderberry extracts (1.74–2.20 mmol TE/g extract and 0.63–0.89 mmol TE/g extract, for ABTS and DPPH radical scavenging, respectively) [29]. 

The phenolic composition of the elderberry polar extract was studied by ultra-high-pressure liquid chromatography-tandem mass spectrometry (UHPLC-MS*^n^*). Phenolic compounds were quantified by UHPLC-UV using calibration curves of reference compounds representative of each chemical family (Table 2). The phenolic compounds identified, as well as their retention time, the maximum UV wavelengths absorption, the corresponding [M − H]^−^ or [M + H]^+^ ions and the key MS*^n^* fragmentation product ions relevant for their identification are summarized in Table 3. Their content is also included and expressed as g of compound/100 g extract. 

Eight phenolic compounds were identified, representing a total of 12.64 g/100 g extract (Table 3), namely: caffeyolquinic acid, cyanidin 3,5-diglucoside, cyanidin 3-sambubioside-5-glucoside, cyanidin 3-glucoside, cyanidin 3-sambubioside, quercetin 3-glucoside, quercetin 3-rutinoside and quercetin. Compounds were identified by comparison of their characteristic retention time and fragmentation patterns obtained under the same experimental conditions, by coinjection of standards or by comparing their MS fragmentation patterns with published data (Table 3).

All the phenolic compounds reported here have been previously reported in elderberries [9,10,11,32,33]. Cyanidin 3-glucoside and cyanidin 3-sambubioside were the major phenolic compounds found in *S. nigra* polar extract, accounting for 4.46 and 4.80 g/100 g extract (368 and 396 mg/100 g fresh berries weight (f.w.)), respectively. These two anthocyanins were previously reported as the major phenolic compounds present in ripe elderberries, accounting for 204–739 mg/100 g f.w., and 122–630 mg/100 g f.w. of cyanidin 3-glucoside and cyanidin 3-sambubioside, respectively [9,10,11,32,33]. Quercetin 3-rutinoside was the major flavonol detected in this extract, accounting for 1.43 g/100 g extract (118 mg/100 g f.w.), being in agreement with published data (up to 96 mg/100 g f.w.) [10].

The overall sugar content of the polar extract was also determined, representing 67 ± 8 g sugars/100 g of extract. As elderberries’ sugar fraction is composed mainly of fructose and glucose [9], it may be inferred that this fraction also contains these sugars. Thus, it is expected that these contribute to the overall energy intake of the rats’ diet.

### 2.2. Elderberry Extract Toxicity Evaluation Using A. fischeri Bioluminescence Model

**Figure 2:**
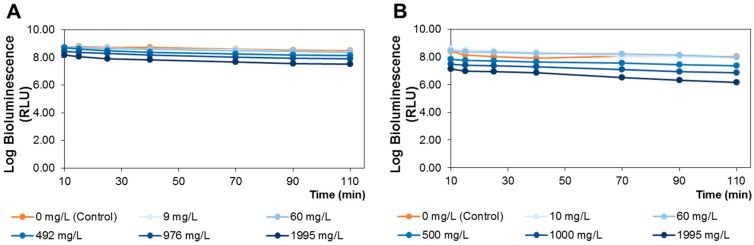
Bioluminescence monitoring of *A. fischeri* treated with elderberry polar (**A**); and lipophilic (**B**) extracts.

In order to evaluate elderberry extract toxicity using *A. fischeri* bioluminescence assay, firstly the correlation between the colony-forming units (CFU) and the bioluminescence signal (relative light units, RLU) of *A. fischeri* was carried out [25]. A linear correlation between CFU and the bioluminescence signal was obtained with *r*^2^ of 0.965 and log_RLU_ = 0.84 log_CFU_ + 0.72, confirming that the bioluminescence reflects the viable bacterial activity. Then, the *A. fischeri* bioluminescence was assessed when exposed to polar and lipophilic elderberry extracts during up to 110 min and at concentrations ranging from 9 to 1995 mg/L (Figure 2). For each extract, a control (0 mg/L) condition was also considered.

For both extracts, at lower concentrations (9–60 mg/L), the bioluminescence signal (expressed as RLU) decreased from 0% to 1.5% in comparison with control (Figure 2), which allowed to infer that these concentration levels were innocuous for *A. fischeri*. Considering the polar extract, for the highest concentration (1995 mg/L), the RLU decreased from 6% to 12% (Figure 2A), unveiling a slight impact in the viable bacterial activity, while for the lipophilic extract, the bioluminescence signal decreased until 23% at the same concentration (Figure 2B). Thus, 60 mg/L was selected, and the corresponding dose to be administrated to rats was calculated by applying the equation of interspecies correlation between *A. fischeri* and rats for oral administration (log*_extract concentration A. fischeri_* = 0.55 log*_extract dose mouse_* − 0.13; also, a mouse to rat dosage conversion factor was used) [34], resulting in a dosage of up to 5194 mg/kg of body weight. Therefore, the diets were prepared ensuring that both extracts’ doses did not exceed this value, and also taking into consideration the relative proportion of the target chemical families in each extract (data from Section 2.1), which corresponded to 190 and 500 mg/100 g of diet of the lipophilic and polar extracts, respectively.

### 2.3. Effect of the Elderberry Extract Supplementation on Dietary Intake, Body Weight and on Blood Morphology and Hematology Indices

The dietary intake, overall growth and organ indices were determined (Table 4). Results indicate that there were no significant differences in food intake in the four experimental groups (*p* > 0.05). The daily extract intake represented 350 and 190 mg/kg b.w. for the polar and lipophilic extracts’ supplementation, respectively. These doses correspond to, 35, 9 and 31–42 mg/kg b.w. in terms of anthocyanin-derivatives, quercetin-derivatives, and triterpenic-derivatives, respectively. For an average person of 70 kg, this would correspond to 397, 102 and 352–477 mg/day of anthocyanin-derivatives, quercetin-derivatives, and triterpenic-derivatives, respectively.

A significant difference (*p* < 0.05) is observed in the body weight variation between non-diabetic (NDB) and the two diabetic groups, diabetic group/not supplemented (DBNS) and diabetic group/supplemented with polar extract (DBPE), while within the STZ-induced diabetic rats (DB groups), no statistical differences were observed (*p* > 0.05). Additionally, no significant changes were observed regarding the body mass/body length ratio (*p* > 0.05). The high-fat (HF) diet and diabetic status had a significant effect (*p* < 0.05) on the rats’ relative kidney mass (% of body weight), of up to 1.4-fold higher than NDB group, being in agreement with previously reported data for HF/STZ-induced diabetic rats [35]. Other tissues such as liver, spleen, heart, testes, pancreas and femur bones were not remarkably affected (*p* > 0.05). The lower body weight variation in STZ-injected rats is probably connected to poor glycemic control, accompanied with a protein catabolism to provide amino acids for gluconeogenesis, that results in muscle wasting and weight loss in diabetic rats [36], while the increase in the weight of kidney (hypertrophy) in proportion to the body weight in STZ-injected rats was suggested to be linked to local alterations in the production of one or more growth factors and/or their receptors in insulin dependent diabetes mellitus [37].

Blood morphological and hematological indices were also determined in order to follow the general health status of the rats during the experiment (Table 5). In fact, the assessment of hematological parameters can be used to reveal the deleterious effects of foreign compounds. Moreover, it may evidence abnormalities in enzymes, metabolic products, hematology, and/or normal functioning of the organs [38].

Most of these indices were not markedly different amongst the four tested groups (*p* > 0.05), however, mean corpuscular hemoglobin concentration (MCHC), white blood cell count (WBC), monocyte count (MONO), lymphocyte count (LYMPH), platelet count (PLT) and red cell distribution width based on standard deviation (RDW-SD) showed significant differences (*p* < 0.05), between at least two groups. In particular, MCHC and RDW-SD values were slightly altered (*p* < 0.05) between NDB and DBPE, and NDB and DBNS, respectively, although the remaining parameters were not altered owing to the red blood cells’ and hemoglobin’s status. Taking into consideration that alterations in red blood cells’ parameters are indicators of anemic status [38], these results suggest the absence of anemic status. The reduction of PLT levels in diabetic rats induced with streptozotocin was confirmed in this study in relation to the non-diabetic rats (NDB), being known that the STZ administration implies abnormalities in the platelets’ function [39]. STZ-diabetes induction (DB groups) led to a decrease in WBC, MONO and LYMPH levels (*p* < 0.05) when compared to NDB rats, as already reported [38]. Streptozotocin suppresses the immune system by damaging WBC and its differentials, such as monocytes and lymphocytes [38].

### 2.4. Effect of the Elderberry Extract Supplementation on Fasting Blood Glucose and Insulin

**Figure 3:**
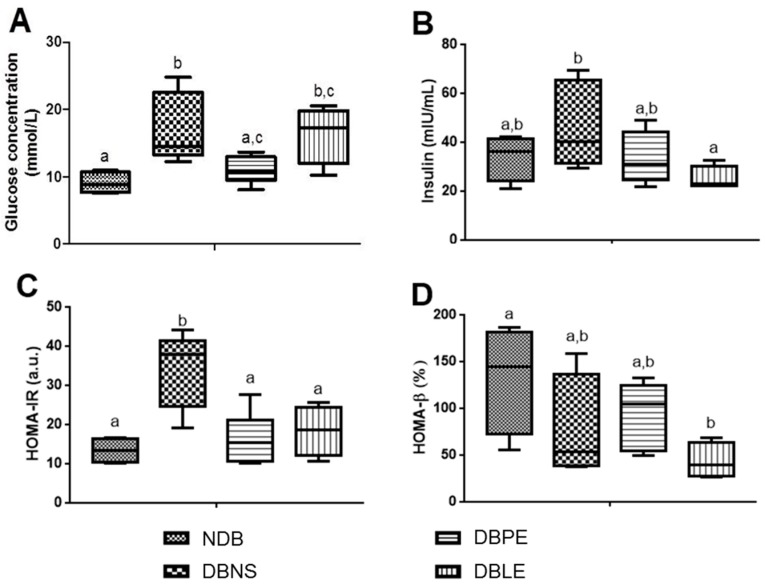
Elderberry extracts dietary supplementation effects on (**A**) fasting blood glucose concentration; (**B**) fasting plasma insulin; (**C**) insulin resistance index (HOMA-IR); and (**D**) β cells function index (HOMA-β) on high-fat diet fed STZ-induced diabetic rats. Diabetic rats without dietary supplementation and non-diabetic rats were also followed-up. NDB: non-diabetic group; DBNS: diabetic group/not supplemented; DBPE: diabetic group/supplemented with polar extract; and DBLE: diabetic group/supplemented with lipophilic extract. Mean values with different letters show statistically significant differences (*p* < 0.05, LSD’s Fisher test).

The levels of fasting blood glucose and insulin levels, as well as insulin resistance and β cells function indices, are presented in Figure 3. After 4 weeks, the STZ injection (DBNS) caused an almost 1.6-fold increase in blood glucose levels (Figure 3A), attaining a median glucose level of 14 mmol/L. Polydipsia and polyuria was observed on the animals in this group, as already reported [40]. After elderberry polar extract supplementation (DBPE), a significant decrease (*p* < 0.05) of the fasting blood glucose was observed when compared to DBNS (Figure 3A), with values similar to the NDB group (*p* > 0.05). Significant reduction in glycaemia levels on STZ-induced diabetes rats after supplementation of the same extract but at lower doses and longer times was already reported (50 mg/kg b.w. during 16 weeks) [5,14]. No significant differences (*p* > 0.05) between the DBNS and the STZ-induced diabetic rats supplemented with lipophilic extract (DBLE) are reported.

Regarding the plasma insulin levels (Figure 3B), it was found that the DBLE group had a lower insulin level (*p* < 0.05) compared to DBNS, being the values similar to the NDB group (*p* > 0.05). For DBPE, the insulin levels did not differ from NDB and DBNS groups (*p* > 0.05). Insulin resistance is a significant feature of diabetes, resulting in the deregulation of carbohydrate metabolism and decreased activity of glycolysis enzymes, which ultimately causes impaired peripheral glucose utilization and augmented hepatic glucose production [40]. Our results suggested an ameliorating effect of elderberry preparations on insulin resistance (Figure 3C), being most likely caused by decreasing fasting blood glucose and by insulin secretion modulation. No significant differences are reported between the two diabetic supplemented groups (*p* > 0.05) (Figure 3C). Regarding β cells’ function index (HOMA-β), our results showed no differences amongst the three diabetics groups (Figure 3D).

### 2.5. Effect of the Elderberry Extract Supplementation on Fasting Lipids, Inflammatory and Toxicity Markers

Sera lipidic markers were assessed in the different groups (Table 6). No significant differences (*p* > 0.05) are reported regarding the total cholesterol, HDL-c and LDL-c, as well as in triacylglycerols, among the four experimental groups. Previous results showed lower levels of total cholesterol, HDL-c, LDL-c, and triacylglycerol in STZ-induced diabetic rats after supplementation with elderberry polar extract (50 mg/kg b.w. during 16 weeks) when compared with non-supplemented diabetic rats [5]. The different diet, administrated dosages and supplementation length may explain these differences.

Renal and hepatic function markers were also assessed (Table 6). Although no significant differences (*p* > 0.05) on the total protein levels amongst the three diabetic groups were observed, higher creatinine and urea levels were observed for DBNS group when compared to NDB group (*p* < 0.05). This suggests alterations in the renal function of diabetic rats after the STZ-induction [36], which is corroborated by the kidney hypertrophy reported in Table 4. Elderberry extract dietary supplementation (DBPE and DBLE) did not show statistical differences of the urea levels compared to DBNS (*p* > 0.05). Regarding the creatinine parameter, DBLE led to a significant decrease being lower than DBNS (*p* < 0.05).

The levels of intracellular enzymes’ alanine aminotransferase (ALT) (particularly found in liver), aspartate aminotransferase (AST) and alkaline phosphatase (ALP) (both enzymes found in different tissues) were also assessed in blood sera. Higher levels of ALP (*p* < 0.05) after diabetes induction with STZ injection (DBNS) were observed, but no statistical differences were found for AST and ALT (*p* > 0.05) when compared with DBNS and NDB. Likewise, no significant differences were reported amongst the three diabetic groups (*p* > 0.05) for the three analyzed enzymes. Enzyme leakage into circulation is an indication of damaged cells due to inflammation or necrosis, this toxicity possibly being induced by STZ administration [41]. Elderberry extract dietary supplementation did not show an additional toxicity as the values did not differ statistically amongst the three diabetic groups (*p* > 0.05).

### 2.6. Effect of the Elderberry Extract Supplementation on Sera and Tissular Zn, Fe and Cu Levels

The status of some trace elements might be disturbed in chronic hyperglycemia [42], and minerals such as Zn and Cu can modulate glucose and lipid homeostasis [43]. Furthermore, the components present in elderberry extracts might interact with these trace elements, which possibly affect in vivo mineral absorption and metabolism [27]. From these perspectives, the tissular and sera mineral status of the rats (Fe, Zn and Cu) was analyzed after 4 weeks of *S. nigra* berry extract dietary supplementation. The results are included in Table 7.

These results indicate that diabetes caused by STZ injection altered the mineral homeostasis of Zn in kidneys, as well as Cu in sera and kidneys (*p* < 0.05). Liver and sera Zn and liver Cu were not altered amongst the four tested groups (*p* > 0.05), neither were tissular or sera Fe (*p* > 0.05). For the cases where mineral homeostasis was changed after STZ-injection, no significant changes were observed after supplementation with the two extracts (*p* > 0.05), which allows inferring that STZ administration had a significant role (*p* < 0.05) in the trace elements’ homeostasis, particularly Zn (kidney) and Cu (kidney and sera), which might be associated with metabolic disturbances occurring in diabetes mellitus [42]. The elderberry dietary extract supplementation did not show a significant additional effect, as the trace element levels did not differ amongst the three diabetic groups in all tested tissues and blood sera (*p* > 0.05).

## 3. Materials and Methods

The experimental setup of this study (Figure 1) includes extracts preparation and partial chemical characterization (phenolic composition by UHPLC-MS*^n^* analysis), evaluation of extract toxicity; animals (Local Animal Bioethics Committee in Poznan, Poland (No 3/2015)—16 January 2015), diet and supplementation; and finally, the biofluid and tissue analysis, as described in detail in the following sections.

### 3.1. Reagents

Methanol (≥99.9%) was purchased from Panreac (Barcelona, Spain). Dichloromethane (≥99.9%) was supplied by Sigma Chemical Co. (Madrid, Spain). Hydrochloric acid (37%, *w*/*w*) was purchased from Riedel-De Haёn, Sigma (Seelze, Germany). 2,2-Diphenyl-1-picrylhydrazyl (DPPH^·^), 6-hydroxy-2,5,7,8-tetramethylchroman-2-carboxylic acid (Trolox, ≥97%), 2,2′-Azino-bis(3-ethylbenzothiazoline-6-sulphonic acid) (ABTS, >98%), cyanidin 3-glucoside chloride (≥95%) and quercetin-3-glucoside (≥98%) were purchased from Sigma-Aldrich (St. Louis, MO, USA). Maltodextrin was purchased from Nowamyl (Łobez, Poland). Nitric acid (65%, *w*/*w*) was purchased from Merck (Darmstadt, Germany). Formic acid (≥98%) was purchased from Fluka Chemie (Madrid, Spain). HPLC-grade methanol and water were supplied from Fisher Scientific Chemicals (Loures, Portugal) and further filtered using a Solvent Filtration Apparatus 58,061 from Supelco (Bellefonte, PA, USA). Reference Bovine Liver, NIST-1577C was purchased from LGC standards (Dziekanów Leśny, Poland).

### 3.2. Elderberry Samples

Elderberries (*S. nigra* L.) were supplied by the Adega Cooperativa do Vale Varosa—RégieFrutas (Tarouca, Portugal). The samples were collected on an experimental field (41.043233° N, 7.728820° W) with 0.5 ha, from 13/14-years old plants, where each plant produces approximately 15 kg of elderberries per year. 

Samples were harvested on the same day between 9 and 12 a.m., in which several bunches from diverse shrubs were randomly harvested and mixed together. Samples were immediately transported under refrigeration (ca. 2–4 °C) to the laboratory and then stored at −20 °C. Prior to extraction, elderberries were freeze-dried using a VirTis BenchTop K (SP Industries, Stone Ridge, NY, USA).

### 3.3. Extract Preparation

The lipophilic extract (LE) was obtained as previously described [13]. Briefly, freeze-dried elderberries (approximately 850 g) were Soxhlet extracted using dichloromethane for 8 h. The solvent was evaporated to dryness in a rotary evaporator and the extracts weighed. This extract was previously chemically characterized by gas chromatography-mass spectrometry analysis [13]. The resulting lipophilic-free solid residue was then extracted (*m*/*v* 1:5) with acidified methanol (0.5% HCl) for one hour under constant stirring, based on previous publications [14,28]. The suspension was then filtered and the extraction process repeated 5 times. The extracts were combined and then evaporated to dryness by low-pressure evaporation. The extract was freeze-dried to ensure the absence of water. As this extract is highly hygroscopic, it was mixed with maltodextrin at a ratio of 1:0.7 (*m*/*m*, extract/maltodextrin).

### 3.4. Phenolic Compound Analysis

#### 3.4.1. UHPLC-MS*^n^* Analysis

UHPLC-MS*^n^* analysis was conducted based on previous methodologies developed in our lab [31], in which the UHPLC system consisted of a variable loop Accela autosampler (200 vial capacity set at 16 °C), an Accela 600 LC pump and an Accela 80 Hz PDA detector (Thermo Fisher Scientific, San Jose, CA, USA). Before UHPLC injection, each extract was dissolved in methanol (HPLC grade), with a concentration of 15 mg/mL, being subsequently filtered with a 0.2 μm PTFE syringe filter. A gradient elution program was carried out for the separation of the analytes, using a Kinetex C_18_ (100 mm × 2.1 mm × 1.7 μm) column supplied by Phenomenex (Torrance, CA, USA), at 45 °C and a flow rate of 0.39 μL/min. The injection volume was 20 μL and the mobile phase consisted of methanol (A) and water:formic acid (95:5, *v*/*v*) (B). It was applied at a linear gradient that consisted of: 0–3 min: 1% A, 3–8 min: 1%–10% A, 8–21 min: 10%–28% A, 21–28 min: 28%–65% A, 28–31 min: 65% A, 31–35 min: 65%–1% A, followed by 4 min of column re-equilibration before the next run. Detection was carried out in the diode array detector (DAD) at 280, 340 and 520 nm, and UV spectra in a range of 210–600 nm were also recorded. A LCQ Fleet ion trap mass spectrometer (Thermo Finnigan, San Jose, CA, USA), equipped with an electrospray ionization source and operating in negative and positive modes was used to perform tandem mass spectrometry analysis. The nitrogen sheath and auxiliary gas were 40 and 10 (arbitrary units), respectively. The capillary temperature was 330 °C and the spray voltage was 5 kV. The capillary and tune lens voltages were set at 41 V and 110 V for positive mode and for negative mode at −36 V and −120 V. CID-MS*^n^* experiments were performed on mass-selected precursor ions in the range of *m*/*z* 100–1500. The isolation width of precursor ions was 1.0 mass units. Collision energy was optimized between 20 and 35 (arbitrary units), using helium as collision gas and scan time was equal to 100 ms. Xcalibur^®^ data system (Thermo Finnigan, San Jose, CA, USA) was used for data acquisition.

Cyanidin 3-glucoside and quercetin 3-glucoside standard solutions (in methanol, with five different concentrations each, between 0.1 and 20 μg/mL), were used for quantification using UHPLC-DAD system. Limits of detection (LOD) and quantification (LOQ) were also estimated using the S/N approach (*n* = 5). Individual compound quantification was accomplished with calibration data for the most similar standards in terms of maximum wavelength absorption, when no pure reference compounds were available. The concentration of each compound was expressed as the mean value (*n* = 3).

#### 3.4.2. Radical Scavenging Capacity

DPPH and ABTS radical scavenging capacities were determined using Lambda 35 spectrophotometer (Perkin-Elmer, Waltham, MA, USA) following previously described procedures [44,45]. The samples were appropriately diluted in methanol. Calibration curves were performed using Trolox as standard, with concentrations between 0.10 and 0.40 mg/mL (*r*^2^ = 0.9946) for ABTS assay and 0.02 and 0.20 mg/mL (*r*^2^ = 0.9955) for DPPH assay. The results are expressed in mmol Trolox equivalents. All determinations were performed in triplicate.

### 3.5. Sugar Content of the Polar Extract

Phenol-sulfuric acid colorimetric method was used to determine the sugar content of the polar extract [46]. It was added to the extract 1 mL of H_2_SO_4_ 72% (*w*/*w*) and 160 μL of phenol 5% (*w*/*w*). The tubes were heated at 100 °C during 5 min, cooled to room temperature and stirred. Absorbance was measured at 490 nm (Lambda 35, Perkin-Elmer) and a calibration curve was prepared using glucose as standard (0–1 mg/mL). The determinations were performed in triplicate. 

### 3.6. A. fischeri Bioluminescence Assay

Toxicity was evaluated through the bioluminescence assay using the bacteria *A. fischeri,* based on a previously established methodology [25]. A bioluminescent marine bacterium *A. fischeri* ATCC 49387 (USA) bacterial strain was used (stored at −80 °C in 10% glycerol). The bioluminescent *A. fischeri* fresh plate cultures were maintained at 4 °C in solid BOSS medium (1% peptone, 0.3% beef extract, 0.1% glycerol, 3% NaCl, 1.5% agar, pH 7.3). One isolated colony was aseptically inoculated in liquid BOSS medium (30 mL), and kept at 26 °C under constant stirring (170 rpm) during 18 h. Then, an aliquot (200 μL) was sub-cultured in BOSS medium (30 mL), and grew at 26 °C under stirring (170 rpm) overnight. The colony-forming units (CFU) and the bioluminescent signal (in relative light units, RLU) correlation of *A. fischeri* was also assessed [25].

For bioassay purposes, an overnight culture of *A. fischeri* was used after a ten-fold dilution in phosphate buffered saline (PBS: 30 g NaCl, 0.2 g KCl, 1.44 g Na_2_HPO_4_ and 0.24 g KH_2_PO_4_ per liter; pH 7.4). For each, 15 mL of bacterial suspension were aseptically distributed in 100 mL acid-washed and sterilized glass beakers containing appropriate amounts of *S. nigra* extract to achieve a final concentration between 0 (control) and 1995 mg of extract/L, respectively. For the lipophilic (dichloromethane) extract, 2% (*v*/*v*) of dimethyl sulfoxide (DMSO) was added in order to dissolve this extract. Previously to the development of toxicity tests, solutions of 2% DMSO were analyzed to check the absence of toxic effects for *A. fischeri*, revealing no toxic effects for up to 110 min. All beakers were wrapped with aluminum foil to protect from light exposure and incubated under 120 rpm stirring at 25 °C. Aliquots, 500 μL, of treated and control samples were collected at time 10, 15, 25, 40, 70, 90 and 110 min and the bioluminescence signal was measured in a luminometer (peak wavelength detected at 420 nm, standard range: 300–650 nm) (Promega Glomax 20/20 luminometer, Turner Designs, Inc., Madison, WI, USA). The tested extract concentrations were selected to have a wide range of concentrations (from 0 to 1995 mg of extract/L), in order to establish the non-toxic doses to be administrated to rats. These doses were calculated based on previously established correlations between the *A. fischeri* bioluminescent model and rat toxicity assays [34,47]. Three independent experiments for each tested condition were done.

### 3.7. Animals, Diets and Elderberry Extract Supplementation

All animal procedures and the protocol were conducted according to EU Directive 2010/63/EU for animal experiments and approved by the Local Animal Bioethics Committee in Poznan, Poland (No. 3/2015). All necessary efforts were made to minimize the number of animals used and their suffering.

Male adult Wistar rats (*n* = 26, 11 weeks old) were purchased from the Licensed Laboratory of the Animal Breeding Center (Poznan, Poland). After arrival at the animal care facility, rats were kept under controlled temperature (21 ± 2 °C) and humidity (55%–60%) with a 12 h/12 h day/night cycle throughout the experiment. After a 5-day adaptation period, animals were divided into 4 groups (initial mean body weight = 330 g), and kept in metal-free individual cages: NDB (non-diabetic group, *n* = 5), DBNS (diabetic group/not supplemented, *n* = 8), DBLE (diabetic group/supplemented with lipophilic extract, *n* = 6) and DBPE (diabetic group/supplemented with polar extract, *n* = 7). Animals were fed ad libitum for 2 weeks: (i) non-diabetic group was fed with semi-synthetic standard composed by casein (14%), sunflower oil (10%), wheat starch (56.5%), sucrose (10%), potato starch (5%), vitamin mix AIN-93M (1%) and mineral mix AIN-93M (3.5%); while (ii) the three diabetic groups (DBNS, DBLE and DBPE) received high fat (HF) diet (40% calories from fat), which were obtained from the basal AIN-93M diet [48], by replacement of wheat starch with fat, being thereby composed by casein (14%), sunflower oil (10%), wheat starch (46.5%), lard (10%), sucrose (10%), potato starch (5%), vitamin mix AIN-93M (1%) and mineral mix AIN-93M (3.5%). Polar extract was incorporated on wheat starch, while lipophilic extract was mixed with sunflower oil. An excessive amount of fat in the diet is one of the factors contributing to insulin resistance in animal models, and thus, the group of rats fed with a high-fat diet was formed to elucidate changes associated with this syndrome [27].

After 2 weeks of controlled diet, the three diabetic groups (DBNS, DBLE and DBPE) were subjected to multiple intraperitoneal injection of STZ freshly dissolved in 0.1 M-citrate buffer (pH 4.4), given in 3 subsequent doses: 20, 10 and 25 mg/kg body weight, in weekly intervals, while NDB group were injected in the same manner, but with the carrier alone (citrate buffer). The approach with multiple doses of STZ combined with a high-fat diet has been shown to be more efficient and stable animal model of diabetes type-2 [49]. The presence of diabetes in rats was confirmed by measuring fasting blood glucose concentration in blood samples (>11 mmol/L) withdrawn from the tail tip after 48 h using a glucometer iXell^®^, Genexo (Warsaw, Poland). After that, dietary supplementation was performed using 500 mg of polar extract/100 g HF diet for DBPE group and 190 mg of lipophilic extract/100 g HF diet for DBLE group. 

All diets were prepared weekly and stored in sealed containers at 4 ± 1 °C. Food intake was measured daily and body mass every 7 days.

After 4 weeks of feeding and overnight fasting, the animals were anesthetized with CO_2_ inhalation and dissected to collect blood and the internal organs. Blood samples were drawn from the heart aorta into Vacutest tubes with plasma coagulant Medlab-Products (Raszyn, Poland), coagulated at room temperature for 20 min, and centrifuged at 4000 rpm. Inner organs (liver, kidneys, heart, spleen, pancreas and testes) and femoral bones were also removed, being washed in a saline solution (0.9% NaCl), weighed and stored at −70 °C. Serum samples were separated and kept in aliquots at −70 °C for biochemical assays.

### 3.8. Biochemical Analyses

Blood morphology and biochemical analyses were conducted in a certified laboratory (Laboratorium Medyczne Synevo, Poznan, Poland).

#### 3.8.1. Blood Morphology 

The Drabkin cyanmethemoglobin method was employed to determine blood hemoglobin (HGB) concentration [50]. The remaining parameters were obtained using the CELLDYN-1700 analytical hematology system [51], analyzing the following parameters: red blood cell count (RBC), hematocrit, mean corpuscular hemoglobin (MCH), mean corpuscular volume (MCV), mean corpuscular hemoglobin concentration (MCHC), red cell distribution width based on standard deviation (RDW-SD), white blood cell count (WBC), monocytes (MONO), lymphocytes (LYMPH), platelets (PLT), mean platelet volume (MPV), platelet distribution width (PDW) and platelet large-cell ratio (p-LCR). For each analyzed parameter, three replicates were performed for each animal.

#### 3.8.2. Blood Biochemical Indices

The serum glucose concentration was determined by the hexokinase method [52], while the total cholesterol, LDL-c and HDL-c levels and triacylglycerol levels were all determined using Olympus AU 560 equipment by the colorimetric methods [53,54,55]. The colorimetric method using Biuret method [56], was used to measure total protein concentration, while the Jaffe kinetic method with picric acid was employed to analyze the creatinine levels [57]. The kinetic method using urease and glutamine dehydrogenase was used to determine urea concentration [57]. Enzyme activities of alanine aminotransaminase (ALT), alkaline phosphatase (ALP) and aspartate aminotransferase (AST) were measured by kinetic methods [58]. Plasma insulin concentration was measured by the RIA method using kits specific for rats, Linco Research (St. Charles, MO, USA) [59]. The efficacy of glucose utilization, insulin resistance and β-cell function was characterized by the homeostasis model assessment (HOMA) indices [60]. For each analyzed parameter, three replicates were performed for each animal.

#### 3.8.3. Trace Element (Fe, Cu and Zn) Status in Blood Sera and Organs

Trace element analysis were based on previous established methodologies [42], in which the rat tissues were digested in spectra pure HNO_3_ (65%, *w*/*w*) in the Microwave Digestion System (MARS 5, CEM). Flame atomic-absorption spectrometry F-AAS method (AAS-3 spectrometer, Zeiss, with BC, Jena, Germany), was used to measure Fe, Zn and Cu concentrations in the mineral solutions. Simultaneous analyses of certified reference material (Bovine Liver, NIST-1577C for tissues (Gaithersburg, MD, USA), HUMASY CONTROL 2 for serum (Randox, London, UK)) were performed to assure the accuracy of quantitative determinations of Fe, Zn and Cu. Water content of the tested organs was determined for the expression of the results on dry basis. Ca. 1 g of each sample was weighed and kept overnight at 105 °C. Zn and Cu concentrations in sera samples were determined by F-AAS after diluting these samples with 0.01% Triton-X100 solution (Merck). The serum Fe concentration was determined by the Guanidine/Iron-Zine method [61,62]. Zn, Cu and Fe were selected as their metabolism might be disturbed in insulin resistance and in diabetic states. Particularly, Fe overload may affect glucose homeostasis, while alterations in Zn and Cu metabolism may increase oxidative damage of cells and exacerbate complications in diabetes [27]. For each analyzed parameter, three replicates were performed for each animal.

### 3.9. Statistical Analysis

All the results presented in the tables are expressed as mean ± standard deviation. One-way analysis of variance (ANOVA) followed by a Fisher’s Least Significant Difference (LSD) test using the GraphPad Prism version 7 for Windows (trial version, GraphPad Software, San Diego, CA, USA), was applied for the obtained results. It was considered statistically significant when *p* < 0.05.

## 4. Conclusions

The lipophilic and polar elderberry extract dietary supplementation effects on STZ-induced diabetic Wistar rats fed with a high-fat regimen were evaluated. Extracts toxicity was first assessed using an *A. fischeri* model, revealing that neither extract significantly altered the viable bacterial metabolic activity at concentrations of up to 60 mg/L. Therefore, by applying the equation of interspecies correlation between *A. fischeri* and rats (oral administration), the resultant daily dietary intake of the lipophilic and polar extracts was 190 and 350 mg/kg b.w., respectively. Elderberry polar extract led to a reduction in fasting blood glucose, while lipophilic extract decreased insulin levels. Furthermore, both extracts lowered insulin resistance, without remarkable alterations in the hematological indices, sera lipidic pattern and the homeostasis of trace elements (Zn, Fe, Cu) from sera and tissues (kidney and liver). The highlighted results are the result of an elderberry polar extract dietary supplementation during a shorter period and at higher doses compared to literature (four-fold shorter length and five-fold higher doses) [5,14,15,16,17,18,19]. Thus, considering this dietary supplementation length, these findings illustrate that a subacute elderberry dietary regimen ameliorated diabetic complications. Regarding elderberry lipophilic extract supplementation, it was tested for the first time, as far as we know. Additionally, the dietary *S. nigra* extract supplementation effects in STZ-diabetic Wistar rats fed with HF diet on trace elements as well as on the insulin status have not been studied previously, as far as we know.

The observed improvement of the studied diabetic indices caused by elderberry extract supplementation demonstrates their potential as suitable substrates for the development of new dietary adjuncts that could help alleviate metabolic disorders in diabetes type 2. To go further with the valorization of these extracts, it is crucial to establish in detail their chemical profile, aiming at extract standardization and by relating the elderberry biological effects with their molecular structures.

## Figures and Tables

**Table 1:** Extraction yield and antioxidant activities of *S. nigra* L. polar berry extract.

Parameter		Values
Extraction yield (%, m/m, d.w.)		58.2 ± 6.3
Antioxidant activity	ABTS ^a^	2.37 ± 0.28
	DPPH ^a^	0.63 ± 0.03

Values expressed as mean ± SD, *n* = 3; d.w.—dry weight; ^a^ expressed in mmol TE/g extract; ABTS: 2,2′-Azino-bis(3-ethylbenzothiazoline-6-sulphonic acid); DPPH: 2,2-diphenyl-1-picrylhydrazyl.

**Table 2:** Calibration data used for the ultra-high-pressure liquid chromatography coupled with ultraviolet detection (UHPLC-UV) quantification of phenolic compounds in *S. nigra* L. polar berry extract.

Compound	λ (nm)	Conc. Range (μg/mL)	Calibration Curve ^a^	*r*^2^	LOD ^b^	LOQ ^b^
					(μg/mL)	
Quercetin 3-glucoside	340	1.00 × 10^−1^—20.0	*y* = 137,729*x* + 112,942	0.994	1.92	6.39
Cyanidin 3-glucoside	520	1.00 × 10^−1^—20.0	*y* = 293,965*x* + 55,885	0.999	7.40 × 10^−1^	2.49

^a^ *y*: peak area; *x*: concentration in μg/mL; ^b^ LOD: limit of detection; LOQ: limit of quantification.

**Table 3:** Phenolic compounds identified in *S. nigra* L. berry polar extract and corresponding MS*^n^* fragmentation profiles.

R.t. (min)	Compound	λ (nm)	[M + H]^+^ (*m*/*z*)	MS^2^ (*m*/*z*)	MS^3^ (*m*/*z*)	[M − H]^−^ (*m*/*z*)	MS^2^ (*m*/*z*)	MS^3^ (*m*/*z*)	Content (g/100 g Extract)	Identification
2.1	Caffeyolquinic acid	280	-	-	-	353	191, 179	-	*tr*	[10]
9.4	Cyanidin 3,5-diglucoside ^a^	514, 278	611	449 ^b^, 287	287	-	-	-	0.11 ± 0.02	[10]
10.9	Cyanidin 3-sambubioside-5-glucoside ^a^	514, 278	743	581, 449 ^b^, 287	287	-	-	-	0.80 ± 0.22	[10]
12.9	Cyanidin 3-glucoside ^a^	514, 278	449	287	-	-	-	-	4.46 ± 0.74	coinjection
13.3	Cyanidin 3-sambubioside ^a^	514, 278	581	449, 287	-	-	-	-	4.80 ± 0.91	[30]
17.9	Quercetin 3-glucoside ^c^	349, 265	-	-	-	463	301 ^b^, 179	179, 151	0.87 ± 0.20	coinjection
18.5	Quercetin 3-rutinoside ^c^	340, 258	-	-	-	609	301 ^b^, 255	179, 151	1.43 ± 0.02	[10]
23.3	Quercetin ^c^	259, 367	-	-	-	301	179, 151	-	0.18 ± 0.06	[31]
								Total	12.64 ± 2.21	

Content values expressed as mean ± SD, *n* = 3; R.t.—retention time; *tr*—trace; MS^2^, MS^3^ − second and third stage of mass spectrometry; Calibration curves used: ^a^ cyanidin 3-glucoside, ^c^ quercetin 3-glucoside; ^b^ Product ions were subjected to further MS^3^ fragmentation.

**Table 4:** Effects of dietary supplementation of elderberry polar and lipophilic extracts on the rats’ overall growth indices. Diabetic rats without dietary supplementation and non-diabetic rats were also followed-up.

Index	Non-Diabetic Rats (*n* = 5)	Diabetic Rats with High-Fat Diets		
		Not Supplemented (*n* = 8)	Polar Extract (*n* = 7)	Lipophilic Extract (*n* = 6)
Diet intake (g/24 h)	20.1 ± 1.1 ^a^	20.9 ± 2.1 ^a^	22.7 ± 2.9 ^a^	20.8 ± 2.2 ^a^
Daily extract intake (mg/kg b.w.)	-	-	350 ± 3	190 ± 3
Average body weight at the beginning/end of experiment (g)	424.56/443.88	383.31/383.38	370.73/368.41	410.77/415.67
Body weight variation (g/24 h)	0.69 ± 0.08 ^a^	0.00 ± 0.34 ^b^	−0.08 ± 0.38 ^b^	0.18 ± 0.65 ^a,b^
Body mass/body length ratio (g/cm)	16.7 ± 1.3 ^a^	14.7 ± 1.3 ^a^	14.3 ± 1.1 ^a^	16.1 ± 1.5 ^a^
Liver (% b.m.)	3.04 ± 0.24 ^a^	3.38 ± 0.32 ^a^	3.39 ± 0.22 ^a^	3.26 ± 0.21 ^a^
Kidneys (% b.m.)	0.60 ± 0.04 ^a^	0.85 ± 0.11 ^b^	0.88 ± 0.11 ^b^	0.78 ± 0.12 ^b^
Heart (% b.m.)	0.28 ± 0.02 ^a^	0.29 ± 0.03 ^a^	0.29 ± 0.02 ^a^	0.30 ± 0.07 ^a^
Testes (% b.m.)	0.93 ± 0.04 ^a^	0.97 ± 0.07 ^a^	0.98 ± 0.15 ^a^	0.61 ± 0.35 ^a^
Pancreas (% b.m.)	0.24 ± 0.04 ^a^	0.26 ± 0.02 ^a^	0.26 ± 0.04 ^a^	0.22 ± 0.04 ^a^
Spleen (% b.m.)	0.15 ± 0.02 ^a^	0.15 ± 0.02 ^a^	0.13 ± 0.01 ^a^	0.14 ± 0.02 ^a^
Left femur (% b.m.)	0.21 ± 0.02 ^a^	0.24 ± 0.02 ^a^	0.25 ± 0.02 ^a^	0.23 ± 0.02 ^a^
Right femur (% b.m.)	0.22 ± 0.01 ^a^	0.25 ± 0.02 ^a^	0.25 ± 0.02 ^a^	0.23 ± 0.03 ^a^

Values expressed as mean ± SD; b.w.—body weight; b.m.—body mass; Mean values with unlike letters in rows show statistically significant differences (*p* < 0.05, LSD’s Fisher test).

**Table 5:** Effects of dietary supplementation of elderberry polar and lipophilic extracts on the rats’ blood morphology and hematology indices. Diabetic rats without dietary supplementation and non-diabetic rats were also followed-up.

Index	Non-Diabetic Rats (*n* = 5)	Diabetic Rats with High-Fat Diets		
		Not Supplemented (*n* = 8)	Polar Extract (*n* = 7)	Lipophilic Extract (*n* = 6)
RBC (10^12^/L)	9.42 ± 0.17 ^a^	9.77 ± 0.46 ^a^	9.88 ± 0.54 ^a^	9.50 ± 0.37 ^a^
HGB (mmol/L)	15.24 ± 0.38 ^a^	15.95 ± 0.56 ^a^	16.13 ± 0.82 ^a^	15.53 ± 0.39 ^a^
Hematocrit (%)	44.64 ± 1.58 ^a^	46.14 ± 2.28 ^a^	44.57 ± 3.01 ^a^	43.28 ± 1.70 ^a^
MCV (10^−15^ L)	47.39 ± 1.32 ^a^	47.30 ± 2.77 ^a^	45.17 ± 2.78 ^a^	45.63 ± 2.82 ^a^
MCH (10^−15^ kg)	16.18 ± 0.23 ^a^	16.40 ± 0.87 ^a^	16.34 ± 0.62 ^a^	16.37 ± 0.42 ^a^
MCHC (10^−2^ kg/L)	34.14 ± 0.55 ^a^	34.71 ± 1.04 ^a,b^	36.23 ± 1.01 ^b^	35.92 ± 1.33 ^a,b^
WBC (10^9^/L)	4.51 ± 1.04 ^a^	2.80 ± 0.98 ^b^	3.03 ± 0.49 ^b^	2.97 ± 0.68 ^b^
MONO (10^3^/μL)	0.44 ± 0.15 ^a^	0.17 ± 0.08 ^b^	0.24 ± 0.06 ^b^	0.21 ± 0.09 ^b^
LYMPH (10^3^/μL)	3.72 ± 1.01 ^a^	1.92 ± 0.53 ^b^	2.35 ± 0.51 ^a,b^	2.38 ± 0.63 ^a,b^
PLT (10^12^/L)	1129 ± 99 ^a^	866 ± 129 ^b,c^	960 ± 114 ^a,c^	855 ± 144 ^b,c^
PDW (10^−15^/L)	9.10 ± 0.44 ^a^	9.54 ± 0.24 ^a^	9.73 ± 0.56 ^a^	10.22 ± 1.52 ^a^
MPV (10^−15^/L)	7.92 ± 0.20 ^a^	8.11 ± 0.29 ^a^	8.04 ± 0.51 ^a^	8.25 ± 0.41 ^a^
p-LCR (%)	10.70 ± 1.60 ^a^	12.13 ± 1.91 ^a^	11.86 ± 3.71 ^a^	13.20 ± 3.25 ^a^
RDW-SD (10^−15^ L)	30.68 ± 1.72 ^a^	35.45 ± 1.96 ^b^	33.01 ± 2.01 ^a,b^	33.58 ± 2.07 ^a,b^

Values expressed as mean ± SD; Mean values with unlike letters in rows show statistically significant differences (*p* < 0.05, LSD’s Fisher test); RBC—red blood cell count; HGB—blood hemoglobin concentration; MCV—mean corpuscular volume; MCH—mean corpuscular hemoglobin; MCHC—mean corpuscular hemoglobin concentration; WBC—white blood cell count; MONO—monocyte count; LYMPH—lymphocyte count; PLT—platelet count; PDW—platelet distribution width; MPV—mean platelet volume; p-LCR—platelet large-cell ratio; RDW-SD—red cell distribution width based on standard deviation.

**Table 6:** Effects of dietary supplementation of elderberry polar and lipophilic extracts on the rats’ sera lipids, inflammatory and toxicity indices.

Index	Non-Diabetic Rats (*n* = 5)	Diabetic Rats with High-Fat Diets		
		Not Supplemented (*n* = 8)	Polar Extract (*n* = 7)	Lipophilic Extract (*n* = 6)
Total cholesterol concentration (mg/dL)	99.34 ± 15.01 ^a^	83.34 ± 17.04 ^a^	90.79 ± 11.16 ^a^	90.92 ± 14.25 ^a^
HDL cholesterol concentration (mg/dL)	72.80 ± 3.19 ^a^	64.69 ± 13.32 ^a^	71.37 ± 10.09 ^a^	71.28 ± 11.25 ^a^
LDL cholesterol concentration (mg/dL)	12.15 ± 8.86 ^a^	4.36 ± 1.89 ^a^	7.13 ± 5.03 ^a^	6.90 ± 5.45 ^a^
Triacylglycerol (mg/dL)	88.44 ± 45.21 ^a^	110.79 ± 77.90 ^a^	65.52 ± 14.91 ^a^	82.50 ± 28.87 ^a^
Total protein (10^−2^ kg/L)	6.86 ± 0.40 ^a^	6.36 ± 0.52 ^a,b^	6.09 ± 0.52 ^a,b^	6.45 ± 0.38 ^a,b^
Creatinine (μmol/L)	34.12 ± 5.25 ^a^	48.51 ± 7.79 ^b^	38.65 ± 8.38 ^a,b^	34.92 ± 6.19 ^a^
Urea (mmol/L)	4.88 ± 0.44 ^a^	10.98 ± 2.61 ^b^	15.03 ± 4.76 ^b^	7.78 ± 3.02 ^a,b^
ALT (U/L)	26.00 ± 10.32 ^a^	53.00 ± 26.38 ^a,b^	69.43 ± 30.54 ^b^	54.67 ± 21.02 ^a,b^
AST (U/L)	78.00 ± 32.60 ^a^	97.43 ± 18.21 ^a^	128.14 ± 69.37 ^a^	104.20 ± 33.21 ^a^
ALP (U/L)	60.20 ± 8.73 ^a^	165.00 ± 53.71 ^b^	152.29 ± 34.75 ^b^	108.67 ± 39.05 ^a,b^

Values expressed as mean ± SD; Mean values with unlike letters in rows show statistically significant differences (*p* < 0.05, LSD’s Fisher test). HDL—high-density lipoprotein; LDL—low-density lipoprotein; ALT—alanine aminotransaminase; AST—aspartate aminotransferase; ALP—alkaline phosphatase.

**Table 7:** Effects of dietary supplementation of elderberry polar and lipophilic extracts on the rats’ sera and tissular Zn, Fe and Cu levels. Diabetic rats without dietary supplementation and non-diabetic rats were also followed-up.

Index	Non-Diabetic Rats (*n* = 5)	Diabetic Rats with High-Fat Diets		
		Not Supplemented (*n* = 8)	Polar Extract (*n* = 7)	Lipophilic Extract (*n* = 6)
Zn (μg/g dry mass)				
Liver	144.69 ± 16.78 ^a^	141.48 ± 9.47 ^a^	138.56 ± 17.36 ^a^	144.50 ± 41.78 ^a^
Kidney	94.63 ± 4.06 ^a^	114.04 ± 12.61 ^b^	120.77 ± 17.81 ^b^	114.04 ± 17.89 ^a,b^
Sera (μg/dL)	133.81 ± 23.56 ^a^	121.27 ± 19.26 ^a^	128.10 ± 21.06 ^a^	121.15 ± 12.55 ^a^
Fe (μg/g dry mass)				
Liver	427.63 ± 51.20 ^a^	487.06 ± 108.45 ^a^	459.61 ± 127.83 ^a^	512.50 ± 189.15 ^a^
Kidney	301.93 ± 33.20 ^a^	353.06 ± 58.20 ^a^	299.16 ± 56.05 ^a^	360.12 ± 48.02 ^a^
Sera (µg/dL)	116.76 ± 14.75 ^a^	141.11 ± 21.25 ^a^	139.77 ± 36.31 ^a^	149.23 ± 22.38 ^a^
Cu (μg/g dry mass)				
Liver	20.08 ± 1.11 ^a^	18.30 ± 2.53 ^a^	17.60 ± 1.57 ^a^	18.63 ± 6.50 ^a^
Kidney	26.87 ± 5.60 ^a^	54.29 ± 18.13 ^b^	59.87 ± 16.94 ^b^	46.55 ± 18.81 ^a,b^
Sera (μg/dL)	120.12 ± 8.14 ^a^	94.50 ± 19.30 ^b^	101.45 ± 16.15 ^a,b^	92.20 ± 9.29 ^b^

Values expressed as mean ± SD; Mean values with unlike letters in rows show statistically significant differences (*p* < 0.05, LSD’s Fisher test).
